# Correlation between Temporomandibular Disorders (TMD) and Posture Evaluated trough the Diagnostic Criteria for Temporomandibular Disorders (DC/TMD): A Systematic Review with Meta-Analysis

**DOI:** 10.3390/jcm12072652

**Published:** 2023-04-02

**Authors:** Giuseppe Minervini, Rocco Franco, Maria Maddalena Marrapodi, Salvatore Crimi, Almir Badnjević, Gabriele Cervino, Alberto Bianchi, Marco Cicciù

**Affiliations:** 1 Multidisciplinary Department of Medical-Surgical and Odontostomatological Specialties, University of Campania “Luigi Vanvitelli”, 80121 Naples, Italy; 2 Department of Biomedicine and Prevention, University of Rome “Tor Vergata”, 00100 Rome, Italy; 3 Department of Woman, Child and General and Specialist Surgery, University of Campania “Luigi Vanvitelli”, 80121 Naples, Italy; 4 Department of General Surgery and Medical-Surgical Specialties, School of Dentistry, University of Catania, 95124 Catania, Italy; 5 Verlab Research Institute for Biomedical Engineering, Medical Devices and Artificial Intelligence, 71000 Sarajevo, Bosnia and Herzegovina; 6 Department of Biomedical and Dental Sciences and Morphofunctional Imaging, School of Dentistry, University of Messina, Via Consolare Valeria, 1, 98125 Messina, Italy

**Keywords:** temporomandibular disorders, posture, TMD, diagnostic criteria for temporomandibular disorders, DC/TMD

## Abstract

Background: Temporomandibular disorders (TMDs) are a series of disorders that affect the muscles and joint. Symptoms include joint pain, muscle pain, and limitation of mouth opening. One of several multifactorial diseases, temporomandibular dysfunction has mostly been linked to five etiological factors: occlusion, trauma, severe pain stimuli, parafunctional activities, and psychological elements, including stress, anxiety, and depression. The position of the human body as it is displayed in space is referred to as posture. Several nerve pathways regulate posture, and through ligaments, TMD and posture affect each other. The purpose of this study is to evaluate the possible correlation between posture and TMD through a meta-analysis of the literature; Methods: A literature search was performed on PubMed, Lilacs, and Web of science, and articles published from 2000 to 31 December 2022 were considered, according to the keywords entered. The term “temporomandibular disorders” has been combined with “posture”, using the Boolean connector AND; Results: At the end of the research, 896 studies were identified from the search conducted on the 3 engines. Only three were chosen to draw up the present systematic study summarizing the article’s main findings. The meta-analysis showed through forest plot analysis a correlation between posture and TMD Conclusions: This literature meta-analysis showed a correlation between posture and TMD. Nerve pathways probably regulate both body posture and mandibular posture. Further clinical studies will be needed to confirm this hypothesis and to indicate the main conclusions or interpretations.

## 1. Introduction

Temporomandibular joint (TMJ), which provides essential biological activities, including chewing and speaking, is one of the most intricate and frequently utilized joints in the human body [1]. Avascular, non-innervated fibrocartilage with a strong capacity for regeneration covers the articular surfaces. The motion of the joint is controlled by the masseter, temporalis muscles, internal pterygoid, the external pterygoid, and the digastric muscle [2]. Temporomandibular disorders (TMD) affect the articulation and masticatory muscles, or both. The following are the most typical symptoms and signs: joint sounds (clicks or crepitus), pre-auricular and/or masticatory muscle soreness, and restrictions or deviation during the mandibular opening. One of several multifactorial diseases, temporomandibular dysfunction has mostly been linked to five etiological factors: trauma, severe pain stimuli [3,4,5], parafunctional activities, and psychological elements, including stress, anxiety, and depression [6,7,8,9,10,11,12,13,14]. The position of the human body as it is displayed in space is referred to as posture. The central nervous system-controlled posture by muscle activation enables modifications due to a sophisticated mechanism integrated by the view and hearing [15]. TMD has ligaments and muscle connections with the cervical area, so these connections have led to speculation that posture problems may influence the development of TMD [16,17,18,19,20]. The masticatory cycles should be balanced since unilateral mastication might throw off the body’s postural equilibrium while standing by creating an imbalance in the neck muscles and anterior muscle chains. The most popular method for studying postural control involves measuring the oscillation of the body while it is in an upright, resting position using a force platform [21]. In patients with TMD, the position of the head is depressed as the masticatory muscles change the position of the jaw. Proprioceptive afferents may exhibit changes in the mandibular position, which may have an impact on gait stability and the center of pressure of the foot. In the case of TMD, there are frequently significant differences between therapists regarding the best kind of occlusal splint to utilize [22]. Occlusal splints, which are frequently employed for TMD-related pain alleviation, appear to perform a significant role in this scenario. In more depth, there are many occlusal splint kinds, such as bite plates, with various indications and purposes. The stabilization splints are hard acrylic appliances that offer TMD sufferers a perfect occlusion temporarily and removable, relieving orofacial pain by loosening masticatory muscles [23]. The stabilization splints may result in a neuromuscular balance, removing posterior interferences and supplying a stable occlusal relationship and centric relation. The relationship between craniometrical posture and TMDs has been studied; however, despite the huge number of studies, clinicians and academics remain unconvinced [24]. There is evidence from certain studies that people with TMDs have altered head and cervical spine posture, while no such link is found in other investigations. The skull, mandible, and cervical spine exhibit neurological and biomechanical connections, generating a functional complex that may be referred to as the “craniocervical mandibular system,” which is related to the cervical area via muscles and ligament [25]. The aim of this literature review with meta-analysis was to evaluate the correlation between posture and TMD. In addition, the meta-analysis performed evaluated the effects of occlusal therapy on posture.

## 2. Materials and Methods

### 2.1. Eligibility Criteria

All documents were assessed for eligibility based on the following Population (including animal species), Exposure, Comparator, and Outcomes (PECO):

(P) Participants consisted of patients. 

(E) Exposure consists of patients with temporomandibular dysfunction treated with occlusal splint therapy.

(C) Comparison consists of patients with TMD not treated with occlusal splint therapy.

(O) The outcome is to evaluate the effectiveness of bite therapy on posture, as the second outcome is to evaluate the interference and correlation between temporomandibular disorders and posture.

The following inclusion criteria were employed for this meta-analysis: (1) randomized clinical trial (RCT); (2) TMD patients treated and evaluated by DC/TMD; (3) diagnosis of myofascial pain, myofascial pain with a limited opening; (4) disc displacement with reduction; (5) disc displacement without reduction with limited opening; (6) disc displacement without reduction without limited opening; and (7) papers published in English.

Exclusion criteria were: (1) full-text unavailability (i.e., posters and conference abstracts); (2) studies involving animals; (3) review article; (4) case reports; (5) lack of effective statistical analysis; (6) degenerative joint disease (osteoarthrosis and osteoarthritis); (7) loss of more than five teeth, with the exception of the third molars; (8) medical history of motor or neurological disorders; (9) facial or head trauma; and (10) orthopedic and orthodontic treatment.

### 2.2. Search Strategy

**Figure 1:**
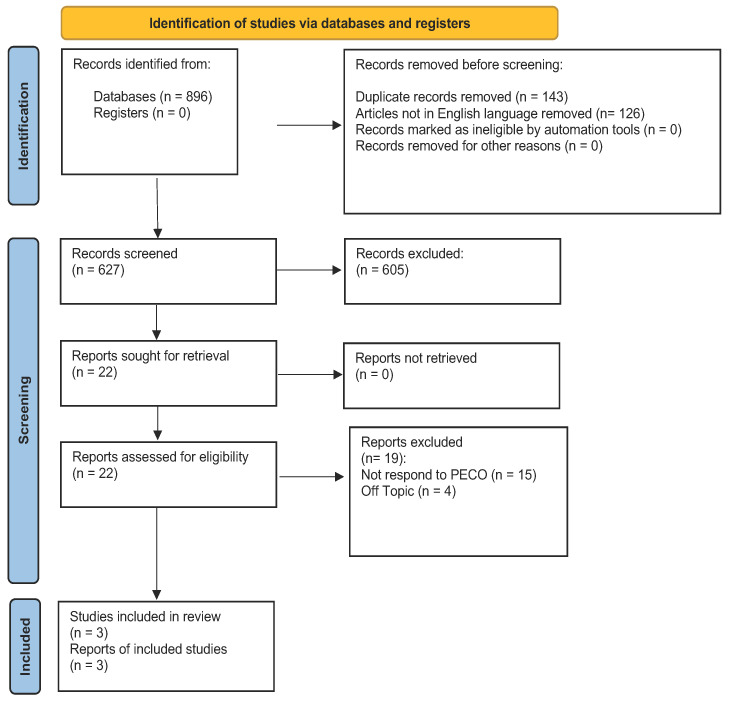
Prisma statement. From: Page, M.J.; McKenzie, J.E.; Bossuyt, P.M.; Boutron l Hoffmann, T.C.; Mulrow, C.D., et al. The PRISMA 2020 statement: an updated guideline for reporting systematic reviews. *BMJ* **2021**, *372*, n71. doi:10.1136/bmi.n71. For more information, visit: http://www.prisma-statement.org/ on 5 March 2023.

A literature search was performed on PubMed, Lilacs, and Web of science, and articles published from 2000 to 31 December 2022 were considered, according to the keywords entered. The term “Temporomandibular disorders” and “TMD” united with OR has been combined with “Posture”, using the Boolean connector AND. The web search was assisted using MESH (Medical Subjects Headings) (Table 1). The criteria for this review are described in the PRISMA and by the following flowchart (Figure 1). Additionally, a manual scan of earlier systematic reviews on the same subject was completed in their references. The Cochrane Handbook for Systematic Reviews of Interventions and Preferred Reporting Items for Systematic Reviews (PRISMA) standards were followed in conducting this systematic review. The International Prospective Register of Systematic Reviews (PROSPERO) has recorded the systematic review procedure under the code CRD42022315350 as of 12 April 2022.

### 2.3. Data Extraction

Using a specialized data extraction on a Microsoft Excel sheet, two reviewers (GM and RF) separately extracted data from the included studies. When there was disagreement, a third reviewer helped to achieve a consensus (MC). 

The following data was obtained: (1) First Author; (2) Year; (3) Sample; (4) Diagnostic criteria; (5) Type of bite; (6) Treatment duration; and (7) Exams to evaluate the effect on posture; (8) Results of therapy.

### 2.4. Quality Assessment

Using the Cochrane risk-of-bias tool for randomized trials, Version 2, two reviewers evaluated the articles’ bias risk (RoB 2). Any discrepancy was discussed with a third reviewer until an agreement was achieved.

### 2.5. Statistical Analysis 

The pooled analyses were carried out utilizing Review Manager 5.2.8 software (Cochrane Collaboration, Copenhagen, Denmark; 2014). The study compared TMD patients receiving biting therapy to a control group to assess the impact on posture. The difference in risk between the two groups was calculated. Low heterogeneity (30%), medium heterogeneity (30–60%), and high heterogeneity (> 60%) were used to measure and categorize study heterogeneity using the Higgins Index (I2) and the chi-square test.

## 3. Results

### 3.1. Study Characteristics 

At the end of the research, 896 studies were identified from the search conducted on the 3 engines. During the initial phase, 143 items were excluded because they were duplicates and 126 because are not in English. During the initial screening phase, 605 articles were excluded from both search engines because they were systematic reviews of the literature, and therefore did not meet the inclusion criteria. In addition, the filter was included in which only randomized clinical trials were considered. During the final screening phase, the abstracts and the full text of 22 articles were evaluated. Only 3 were chosen to draw up the present systematic study, as illustrated by the PRISMA 2020 flowchart in Figure 1; 19 articles were excluded; 15 did not meet PECO; and 4 are off topic. The remaining articles were selected for the title and abstract screening according to the PECO model. The studies considered have a time frame from 2014 to 2018. The studies analyzed were conducted in various parts of the world: Brazil, Italy, and Poland. A total of 154 subjects were analyzed. De Giorgi’s study analyzed 45 women with TMD, evaluated by DC/TMD; however, the patients were all female, and all had joint dislocation with or without reduction. The pain was assessed by VAS scale and the effects of bite therapy on posture were by cephalometric examination and by rasterstereography, after which postural parameters were assessed at time 0 and after 1 month and 3 months. The patients were randomly divided between 24 in the study group and 21 in the control group. Patients in the study group were fitted with a 2 mm splint with posterior contact. Oliveira’s study evaluated 49 patients with TMD assessed through DCs/TMDs, in which case he selected and evaluated all TMD subgroups. The patients were randomly divided as follows: 36 patients in the study group to whom a splint was applied to be worn more than 8 h a day for a duration of 12 weeks; a control group of 13 patients to whom nothing but physiotherapy was applied. After that, postural parameters were evaluated through a stabilometric platform, and the parameters were assessed with eyes closed and open. It was mainly evaluated through speed to re-establish the Center of Pressure (COP). A 1.5 mm splint with simultaneous bilateral contacts was used in this study. The WalczyNska-Dragon study took 60 patients with TMD, diagnosed with DC/TMD. The patients considered in this study had TMD and neck pain with movement limitation. The patients were randomly divided as follows: 30 patients in the study group to whom a splint was applied; and 30 patients as the control group. Postural parameters were assessed by a Jaw motion tracking (JMT) which evaluated mandibular movements and a cervical spine motion (CMS), which evaluated improvements at the cervical and postural levels. In addition, during therapy, pain level was reported and assessed by the VAS scale. Patients were evaluated at time 0, and after 3 weeks and 3 months. The type of appliance was a plate with anterior contacts only.

### 3.2. Main Findings 

The purpose of De Giorgi’s study was to assess how an occlusal splint affected individuals with intra-articular temporomandibular joint (TMJ) problems’ body posture. The study included 45 women with TMJ disorders who were divided into two groups: those who used occlusal splints and those who did not. Rasterstereographic recordings were made at baseline, 1 month, 3 months, and 6 months later in order to examine the following postural parameters: pelvic tilt and torsion, kyphotic and lordotic angles, lumbar and cervical arrows, trunk imbalance, and trunk inclination. In the intragroup analysis, no significant differences were found for the postural parameters. Significant differences between the two groups were found when the cervical arrow, kyphotic angle, and lordotic angle were analyzed. There were no discernible differences between T0 and T1, or T0 and T2 in the postural characteristics of the occlusal splint and control group in the intragroup study. The occlusal splint group between T0 and T3 showed no significant differences either. Significant variations between the two groups were found in the analyses for various postural measures. At T1, the cervical arrow evaluation revealed a statistically significant difference between the control group and the occlusal splint group (61.96 mm 17.73 vs. 58.39 mm 13.73; *p* = 0.001). In terms of the kyphotic angle in the resting position, there was a statistically significant difference at T1 between the control group and the occlusal splint group (54.17o 8.97 vs. 55.25o 10.16; *p* = 0.012), and at T2 between the control group and the occlusal splint group (54.00 vs. 54.84). In terms of the lordotic angle a statistically significant difference was discovered between the control group and the occlusal splint group at rest position at T2 (49.30) (*p* = 0.017) [26]. The study of Oliveira was to investigate how using an occlusal splint influenced postural balance. A prospective, controlled, randomized clinical trial was carried out. A questionnaire developed by the R DC/TD and magnetic resonance imaging of TMJ was used to diagnose 49 patients—36 in the test group and 13 in the control group—who ranged in age from 18 to 75 and were of both sexes. Orientations for physiotherapeutic exercises and an occlusal splint were given to the test group, while just physiotherapeutic exercises were given to the control group. A force plate was used to assess postural balance. The groups were reevaluated after 12 weeks. With their eyes closed, patients from both groups showed a statistically significant increase in antero-posterior speed: test group (*p* 0.001) and control group (*p* = 0.046). With their eyes open, only patients in the test group showed a statistically significant increase in antero-posterior speed (*p* = 0.023) [27]. The purpose of this study was to assess how TMD therapy affected the cervical spine’s range of motion (ROM) and spinal pain relief. Sixty individuals with TMD, cervical spine discomfort, and restricted cervical spine range of motion made up the study group. The subjects completed a questionnaire that asked about their neck pain and TMD symptoms. They also had their masticatory motor system physically evaluated (in accordance with RDC-TMD) and analyzed by a JMA ultrasound instrument. An MCS device was used to analyze the mobility of the cervical spine. The whole group displayed cervical spine discomfort. The treated group’s cervical spine pain on the VAS scale considerably decreased over the course of three months of therapy. Cervical spine pain decreased during therapy; it returned after three weeks for 39% of patients, and it was only present in 8% of subjects from the treated group after 3 months (2 subjects). A statistically significant difference existed between the treatment and control groups (*p*= 0.0001). The flexion movement, which only 22% of patients had on the first assessment, was within normative values, and was where the progress could be detected. A total of 70% of the patients in the treated group’s flexion movement adhered to the standard at the third examination. There were more subjects in the experimental group who had anteflexion movement outcomes that were consistent with the norm, which was very significant (*p* = 0.0006). The results for the retroflexion movement improved by a highly significant factor (*p* = 0.0082); more subjects in the experimental group had results that were in line with the norm. No significant (*p* > 0.05) improvements were identified in the control group, indicating that the cervical spine’s range of motion did not improve in relation to normative values [28] (Table 2).

### 3.3. Metanalysis

**Figure 2:**
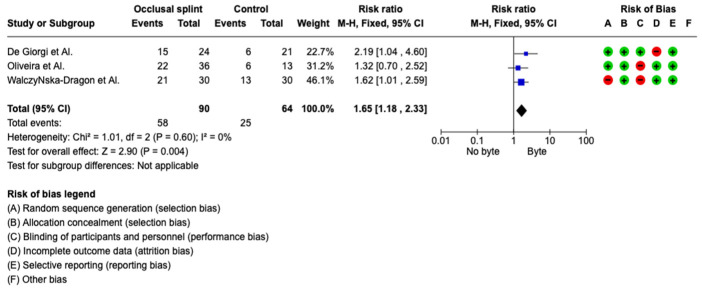
Forest plot of the meta-analysis [26,27,28].

The meta-analysis was conducted by fixed model effect because of the low heterogeneity (*I*^2^ = 0%) between the three included studies. The overall effect, reported in the forest plot (Figure 2), shows that showed that bite therapy and TMD are related to posture and that a change in chewing causes effects on posture (RR 1.65; 95%; CI 1.18–2.53).

### 3.4. Quality Assessment and Risk of Bias

**Figure 3:**
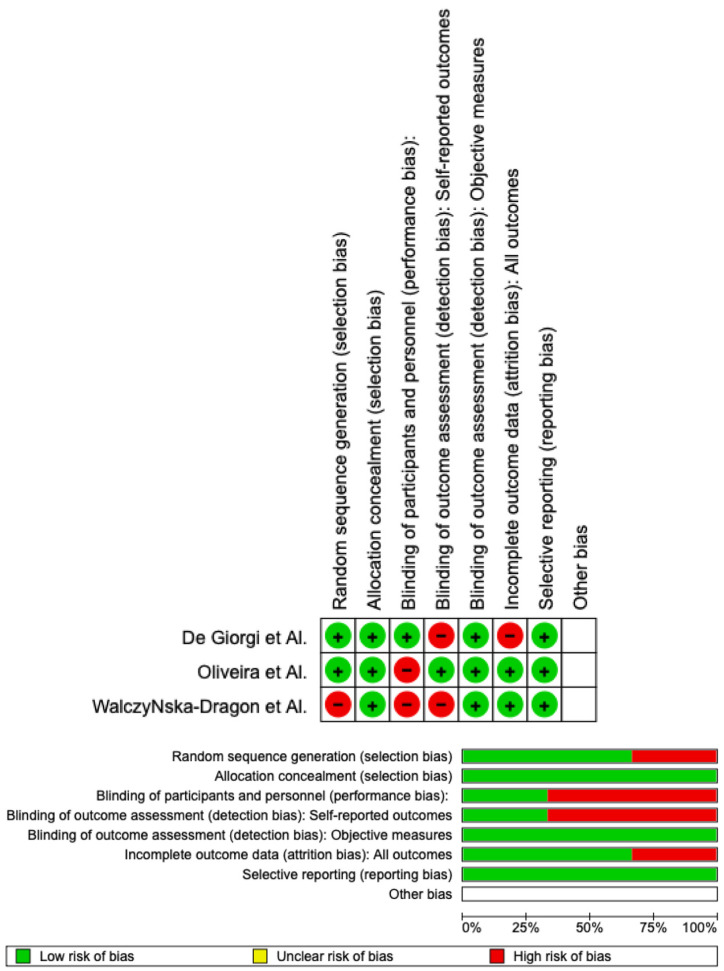
Risk of bias domains of the included studies [26,27,28].

Using RoB 2, the risk of bias was estimated and reported in Figure 3. Regarding the randomization process, 75% of the studies ensured a low risk of bias. However, 25% of the studies excluded performance bias, but 75% reported all outcome data, and 100% of the included studies adequately excluded bias in the selection of reported outcomes, while 25% excluded bias in self-reported outcomes. Overall, all studies were shown to have a low risk of experiencing bias.

## 4. Discussion

The findings and their interpretation in light of prior research and the working hypotheses should be discussed by the authors. It is important to discuss the results and their implications in the widest context possible. It may also highlight potential directions for future study [29].

There is still disagreement in the literature on the relationship between TMJ, muscle and posture, and it is suggested that better controlled trials with thorough TMD diagnoses, larger sample sizes, and objective posture measurements are required [30]. Important gaps in our understanding of this relationship remain as a result of the intricacy of the contributing components. The connection between posture and the TMJ is supported by certain research in the literature, but not all of them [31]. The link between TMD myogenic and posture is heavy but weak between TMD arthrogenus and another type of TMD [32]. De giorgi’s study evaluated through different postural analyses their change. In particular, the use of posturography as a diagnostic tool was not supported by the evidence because these analytic techniques did not make any noteworthy advancements. A dependable, non-invasive technique for examining 3D spine anatomy is rasterstereography. This method enables a radiation-free assessment of the body’s back surface and has proven to be accurate for determining pelvic and spinal alignment. The intragroup analyses revealed no statistically significant differences, proving that treatment with an occlusal splint had no effect on patients’ postural measures. At T1 and T2, however, some variations between the control and occlusal splint groups were discovered, suggesting that some alterations had taken place. However, the results of De Giorgi were not noteworthy from a therapeutic perspective due to the low range of statistical significance [26]. Oliveira et al. [27] found that both with eyes open and closed, the use of an occlusion splint, and a presentation of therapeutic exercises significantly increased the anteroposterior velocity from the COP [33]. In synthesis, the occlusion splint added to the effects of postural control. Studying TMJD and postural balance requires keeping track of patients’ anthropometric and clinical parameters. Age, sex, weight, and height are variables that may have an impact on the effectiveness of the treatment and the postural balance. Weight and height did not significantly differ across the groups. Additionally, there was no difference in terms of age, despite the fact that a higher percentage of TMJD patients were between the ages of 20 and 40. There is a significant amount of research supporting the signs and symptoms of TMD, thus they might not differ from those discovered in our study. An attempt at postural readjustment can be concluded from the findings of the use of an occlusal splint and therapeutic exercise guidelines in subjects with TMD over the course of 12 weeks, which included an increase in the velocity of center gravity recovery. It is possible to interpret the acceleration of the postural sway as an increase in the frequency of postural balance corrections made by the individuals [34,35,36,37,38,39,40,41]. The adjustment of the head and neck posture carried on using the occlusal splint may have caused this increase in the frequency of corrections. The new question is whether these findings point to a transient shift in how the body is perceived, indicating that postural balance will change in the future, or perhaps revert to its initial state.

The results obtained by WalczyNska-Dragon have shown a correlation between the diseases and the beneficial effects of treatment for cervical pain, even in people experiencing such discomfort for a long time. In order to treat patients more effectively and efficiently at the beginning, when painful symptoms first appear, and when treating them is possible and much quicker and more efficiently, it is crucial to understand the intricate relationships between posture and TMD. The cause of cervical spine pain is frequently yet unknown, but from these studies, they seem to be related. Numerous scientific studies support the efforts of many researchers to investigate the effects of problems in the “upper quarter” on posture and pain in different body regions. However, the main goal of studies undertaken thus far has been to demonstrate the existence or lack of a relationship between cervical spine discomfort and stomatognathic system dysfunction. The most often used method involved questionnaires with questions on complaints of the motor aspect of the stomatognathic system and pain in the cervical spine. Based on this, researchers would hunt for a connection between the cervical spine pain and the dysfunction of the stomatognathic system’s motor component. However, in our investigation, occlusal appliance therapy was used; no intrusive treatment strategies were employed. Most of the participants in the experimental group who received therapy with an occlusal splint reported improvements and the complete removal or significant reduction in cervical spine discomfort and TMD symptoms, while the cervical spine’s mobility also increased significantly. When treating TMD, there are frequently significant differences between therapists regarding the best kind of occlusal splint to utilize [28].

Additionally, studies have shown a connection between posture and TMDs. TMD patients have more pronounced alterations in the body’s center of gravity. Numerous investigations have demonstrated that patients with TMD frequently exhibit an overly forward head position that is accompanied by shortening of the sternocleidomastoid and the posterior cervical extensor muscles [42,43]. When the head is tilted anteriorly, the field of vision is reduced, and cervical lordosis rises to extend the field of view. The center of gravity is similarly affected by the head’s anterior position, supporting the link between TMD and body posture. The cervical region’s postural changes can also lead to TMD by altering the position of the mandible and the head’s orientation. Previous posturography studies have shown that in participants with unilaterally anaesthetized trigeminal afferents, there is a link between body posture and gaze balance [44]. According to the findings mentioned above, the trigeminal system has a significant impact on how posture and sight are coordinated. The hypothesized neurological basis is still being developed; however, there are several hypotheses [45]. There have been many documented anatomical links between trigeminal systems and the neurological systems that regulate posture. The mesencephalic nucleus of the trigeminus (MNT), a sensory nucleus with distinctive properties, stretches from the caudal region of the superior colliculus to the dorsal portion of the spinal trigeminal nucleus. These nucleus cells are protoneurons with ganglionic cell functions rather than central neurons [46]. 

The presence of muscle-fascial chains is another fundamental component of the relationship between the stomatognatic system and human posture. Organs are held in place, protected, and fed by fasciae. Fasciae have three layers: superficial, deep, and visceral. Myofibroblasts and several different receptors are heavily concentrated in the deep fasciae, which surrounds muscles, bones, nerves, and blood vessels (nociceptors, proprioceptors, mechanoreceptors, chemoreceptors, and thermoreceptors). Fascial cells called myofibroblasts are produced in response to mechanical stress and actively contract smoothly and resemble muscles [47]. The fascial system contains mechanoreceptors that regulate contractile capacity and can regulate it when stressed, and therefore, distribute muscle tension to adjacent muscles. These tensions move along the muscle fascial chain and affect how the body is positioned in general [48,49]. All these analyzed studies, although they treated patients with different types of splints, showed an intercorrelation between temporomandibular disorders and posture, both in terms of pain and posture [50,51]. All these studies used different methods to be able to evaluate the effects of bite therapy on posture and cervical movements, and this turns out to be a confounding factor in the performed meta-analysis. However, based on the results obtained, there are effects of bite treatment on posture compared to untreated patients, and this shows an intercorrelation between posture and temporomandibular disorders. In addition, a limitation of the study is given by De Giorgi, as he recruited only patients with TMD who have joint disc dislocation. In fact, the limitations of these articles are because the change or readjustment of posture after completing bite therapy is not evaluated, and therefore, the effects over time of this therapy on posture are not evaluated.

## 5. Conclusions

This meta-analysis and review of the literature showed the correlation between posture and temporomandibular disorders. In fact, in a statistically significant manner, the application of bite therapy had positive effects on what is posture. Although with the limitations of these analyzed studies, we can say that this correlation exists and is present.

## Figures and Tables

**Table 1:** Search strategy.

PubMed ((temporomandibular disorders) OR (TMD)) AND (POSTURE)
Lilacs temporomandibular disorders [Palavras] or tmd [Palavras] and posture [Palavras]
Web of Science ((ALL=(temporomandibular disorders)) OR ALL=(tmd)) AND ALL=(posture)

**Table 2:** Principal elements of the studies which formed part of the present systematic analysis.

Author	Year	Sample	Diagnostic Criteria	Type of Byte	Treatment Duration	Exams to Evaluate Effect on Posture	Results
De Giorgi et al. [26]	2020	45 women: 24 test 21 control	DC/TMD	2 mm with posterior contact	1, 2, 3 months	Evaluation of VAS Rasterstereography Cephalometric analysis	Significant differences concerning the cervical arrow, the kyphotic and lordotic angles
Oliveira et al. [27]	2019	49 patients: 36 test 13 control	DC/TMD	1.5 mm with simultaneous bilateral contact	12 weeks	Stabilometry test with the eye open and closed	Study group had increased anteroposterior velocity with eyes closed and eyes open
WalczyNska-Dragon et al. [28]	2014	60 patients: 30 test 30 control	DC/TMD	Byte only with anterior contact	Evaluation after 3 weeks and 3 months	Evaluation of VAS Evaluation of mandibular movement with JMT Evaluation of cervical spine movement with MCS	Test group: improvement in TMJ movement and cervical spinal movement Diminutions of VAS

## Data Availability

All data described in the study are presented in the manuscript. The datasets analyzed are available from the corresponding author on reasonable request.

## Abbreviations

TMD	temporomandibular disorders
TMJ	temporomandibular joint
PECO	patients, exposure, comparison, outcome
DC/TMD	diagnostic criteria for temporomandibular disorders
RCT	randomized clinical trial
MeSh	Medical Subject Headings
ROM	range of motion
